# MicroRNAs, DNA Damage Response, and Cancer Treatment

**DOI:** 10.3390/ijms17122087

**Published:** 2016-12-12

**Authors:** Mingyang He, Weiwei Zhou, Chuang Li, Mingxiong Guo

**Affiliations:** Hubei Key Laboratory of Cell Homeostasis, College of Life Sciences, Wuhan University, Wuhan 430072, China; 2014202040110@whu.edu.cn (M.H.); zhouww@whu.edu.cn (W.Z.); lic93@whu.edu.cn (C.L.)

**Keywords:** microRNAs, DNA damage response, DNA repair, radiotherapy, chemotherapy

## Abstract

As a result of various stresses, lesions caused by DNA-damaging agents occur constantly in each cell of the human body. Generally, DNA damage is recognized and repaired by the DNA damage response (DDR) machinery, and the cells survive. When repair fails, the genomic integrity of the cell is disrupted—a hallmark of cancer. In addition, the DDR plays a dual role in cancer development and therapy. Cancer radiotherapy and chemotherapy are designed to eliminate cancer cells by inducing DNA damage, which in turn can promote tumorigenesis. Over the past two decades, an increasing number of microRNAs (miRNAs), small noncoding RNAs, have been identified as participating in the processes regulating tumorigenesis and responses to cancer treatment with radiation therapy or genotoxic chemotherapies, by modulating the DDR. The purpose of this review is to summarize the recent findings on how miRNAs regulate the DDR and discuss the therapeutic functions of miRNAs in cancer in the context of DDR regulation.

## 1. Introduction

### 1.1. DNA Damage Response (DDR) and Cancer

Each of the ~10^13^ cells in the human body is assaulted by tens of thousands of DNA lesions per day [1]. In addition to normal cellular metabolic processes, many physical and chemical agents around us can produce DNA damage. For example, ultraviolet (UV) light and ionizing radiation (IR) are typical physical genotoxic agents and can induce up to 10^5^ DNA lesions (pyrimidine dimers and (6-4) photoproducts) per cell per day [2]. IR in sunlight or therapeutic radiation can induce single-strand breaks (SSBs) and double-strand breaks (DSBs) in the backbone of the DNA double helix. Other factors, chemical agents, can cause a variety of forms of DNA damage by inhibiting topoisomerase I and topoisomerase II activity or attaching alkyl groups to bases. These chemical agents are generally present in environmental pollutants (such as smoke, factory fumes, and vehicle exhaust) as well as chemotherapeutic agents (e.g., cisplatin and bleomycin).

In order to confront a variety of DNA lesions and ensure genomic stability, cells trigger the DDR to recognize, transduce, and take measures to attenuate DNA damage. The DDR is a complex network that includes specialized sensor proteins to recognize DNA damage, and transducer proteins to recruit subsequent effector proteins responsible for cell cycle arrest, apoptosis, transcription arrest, and DNA repair [3]. Further, confronted with various DNA lesions, cells will select different DNA repair patterns. For example, nonhomologous end joining (NHEJ) and homologous recombination (HR) are two mechanisms of DSB repair.

The DDR is a double-edged sword with regard to cancer development and therapy. On the one hand, because genomic instability is one of the most pervasive conditions underlying onset of tumorigenesis [4], DNA lesions on oncogenes or genes involved in cancer-related processes can initiate tumorigenesis. An impaired DDR that fails to repair DNA lesions may promote tumorigenesis and tumor development. On the other hand, the outcome of the DDR sabotages the work of chemo- and radiotherapy [4].

### 1.2. Biogenesis and Function of MicroRNAs (miRNAs)

**Figure 1:**
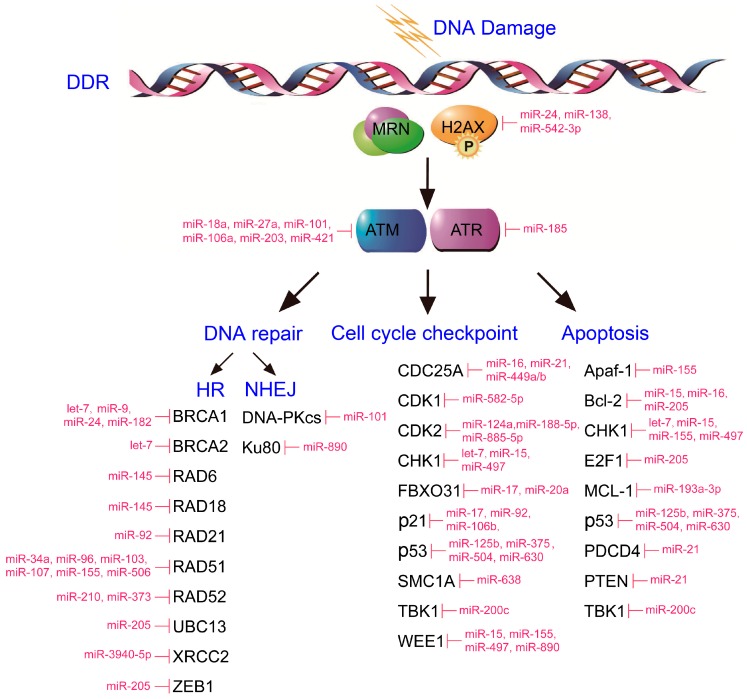
Schematic diagram representing chromosomal DNA and the major components of the DDR signaling pathway (**top**). Major and minor components of the DDR pathway are arranged in columns based on the aspect of the DDR in which the proteins participate (**bottom**). miRNAs that interact with individual DDR components are shown in magenta. (1) miRNAs affect DDR sensors: miR-24 [18], miR-138 [20] and miR-542-3p [20] target H2AX; (2) miRNAs affect DDR mediators: miR-421 [22], miR-101 [23], miR-203 [24], miR-18a [25], miR-106a [25] and miR-27a [26] target ATM; and miR-185 [27] targets ATR; (3) miRNAs affect DNA repair by homologous recombination (HR): miR-9 [28], miR-24 [29], miR-182 [30] and let-7 [31] target BRCA1; let-7 [31] targets BRCA2; miR-145 [32] targets RAD6 and RAD18; miR-34a [33], miR-506 [34], miR-107 [35], miR-103 [35], miR-96 [36] and miR-155 [37] target RAD51; miR-92 [38] targets RAD21; miR-210 [39] and miR-373 [39] target RAD52; miR-205 [40] targets ZEB1 and UBC13; and miR-3940-5p [41] targets XRCC2; (4) miRNAs affect DNA repair by nonhomologous end joining (NHEJ): miR-101 [23] targets DNA-PK; and miR-890 [42] targets Ku80; (5) miRNAs affect cell cycle checkpoint: miR-15 [43], miR-497 [44] and let-7 [31] target CHK1; miR-630 [45], miR-504 [46], miR-125b [47] and miR-375 [48] target p53; miR-106b [49], miR-17 [38] and miR-92 [38] target p21; miR-124a [50], miR-885-5p [51], and miR-188-5p [52] target CDK2; miR-582-5p [53] targets CDK1; miR-200c [54] targets TBK1; miR-17 [55] and miR-20a [55] target FBXO31; miR-15 [43], miR-155 [43], miR-497 [44], and miR-890 [42] target WEE1; miR-16 [56], miR-21 [57], and miR-449a/b [58] target CDC25A; and miR-638 [59] targets SMC1A; and (6) miRNAs affect apoptosis: miR-15 [43], miR-497 [44], let-7 [31], and miR-155 [43] target CHK1; miR-630 [45], miR-504 [46], miR-125b [47], and miR-375 [48] target p53; miR-205 [60] targets E2F1 and BCL-2; miR-16 [61] and miR-15 [62] target BCL-2; miR-193a-3p [63] targets MCL-1; miR-155 [64] targets Apaf-1; miR-200c [54] targets TBK1; and miR-21 [65] targets PTEN and PDCD4. MRN: MRE11/RAD50/NSB1 complex.

Since Victor Ambros and colleagues discovered that miRNA lin-4 had antisense complementarity to multiple sites in the 3′-untranslated region (UTR) of the *lin-14* gene and reduced the level of LIN-14 protein without a change in expression of lin-14 mRNA, studies of the regulatory mechanism and functions of these short noncoding RNAs have been carried out by more and more researchers [5,6]. The mature miRNAs are about 18–25 nucleotides in length, and their biogenesis consists mainly of transcription by RNA polymerase II/III and processing/maturation by two evolutionarily conserved RNase III enzymes, Drosha and Dicer [7,8]. The majority of miRNAs are transcribed by RNA Pol II as primary miRNAs (pri-miRNAs). Following transcription, the Drosha-DGCR8 microprocessor recognizes and processes pri-miRNAs in the nucleus [9]. This step will generate an approximately 70-nucleotide length precursor miRNA (pre-miRNA), which has atypical hairpin structure. Next, the pre-miRNA is translocated from the nucleus to the cytoplasm by exportin-5, and additional processed by Dicer in cytoplasm [10]. By this step, a mature miRNA is yielded, consisting of functional guide strand and passenger strand [11]. The regulatory functions of miRNAs are accomplished through the RNA-induced silencing complex (RISC). miRNA assembles onto RISC, acting as a guide by base-pairing with target mRNA to negatively regulate mRNA expression [12]. By base-pairing at the 3′-UTR, the coding sequence, and 5′-UTR of the mRNA, miRNAs negatively regulate the stability and translation of mRNA. Owing to the vast number of miRNAs and their multiple target genes, the regulatory functions of miRNAs exist in almost all cellular processes, including proliferation, apoptosis, differentiation, senescence, and cell cycle arrest. A large number of genes in the DDR process are targeted and regulated by miRNAs, and several review articles have described the role of miRNAs in regulating the DDR [13,14,15]. In the following section, we will describe some miRNA-regulated genes and the multiple diverse functions of miRNAs in the DDR processes (Figure 1).

## 2. miRNAs Mediate DDR Regulation

### 2.1. Sensors/Mediators/Transducers of DDR

The DDR is a molecular mechanism that cells have evolved to sense DNA damage, transduce these signals and promote their repair [16]. Acting as a sensor of DSB signaling, the MRE11/RAD50/NSB1 (MRN) complex recruits DDR-related proteins, including ataxia telangiectasia mutated (ATM) and other DDR mediators, to the DSB sites [13,17]. ATM-dependent phosphorylation of histone variant H2AX-γH2AX serves as a platform for recruitment of additional DDR factors and enhancement of signaling pathways [17]. In 2009, Lal and colleagues showed that inhibition of H2AX expression and DNA repair in terminally differentiated blood cells is mediated by upregulated miR-24 and also that the miR-24-mediated H2AX suppression rendered hematopoietic cells hypersensitive to DNA-damaging agents [18]. Overexpressed miRNA-138 was also shown to regulate the DDR by inhibiting expression of its target, H2AX, and reducing formation of foci of phosphorylated H2AX [19,20].

Upon the recognition of DNA lesions by sensor/mediator proteins, transducers, such as ATM, ataxia telangiectasia and Rad3 related (ATR), and DNA-dependent protein kinase catalytic subunit (DNA-PKcs) relay, transduce, and amplify the original damage signal to effectors in downstream pathways, including the DNA repair, cell cycle checkpoint, and apoptosis pathways [13]. Some studies have shown, using target prediction programs, that several miRNAs can suppress ATM expression by targeting the 3′-UTR of ATM transcripts. Overexpressed miR-18a in breast cancer cells suppresses ATM expression and its formation of nuclear foci by its downstream substrates H2AX and 53BP1, which reduced the DNA damage repair capacity of cells to irradiation-chemotherapy [21]. miR-421 has also been described as negatively regulating ATM expression, leading to clinically manifest tumor radiosensitivity [22].

On account of multiple miRNAs involved in regulating the expression of important targets in the DDR signal transduction, which subsequently affects cell cycle arrest and other process, these findings may provide new potential therapeutic targets in tumor therapy.

### 2.2. Effectors of DNA Repair

Multiple DNA-repair mechanisms are necessary to repair the wide variety of DNA-lesion types. Under normal physiological conditions, six major DNA-repair pathways counter DNA lesions: base excision repair, mismatch repair, nucleotide excision repair, translesion DNA synthesis, NHEJ, and HR [4]. Because DSBs are among the most toxic DNA lesions, this type of lesion plays the most important role in cancer therapy, which exploits the toxicity of many chemotherapeutic and radiotherapeutic agents to induce DSBs [17]. NHEJ and HR are the two major DSB repair pathways and function in different phases of the cell cycle.

NHEJ is the major pathway for the repair of DSBs because it can function throughout the cell cycle and not require a homologous chromosome. When DSBs occur, the heterodimers Ku (Ku70/Ku80) rapidly binds to breaks. Ku is capable of interacting with the nuclease, the polymerases and the ligase and acts as a tool belt protein that can stabilize any of a number of enzymatic activities at a DNA end [66]. Knockdown of miR-124 in rats has been proved that can protect against neuronal death and apoptosis by targeting Ku70 [67]. Furthermore, it has recently been shown that Ku80 is overexpressed in lung cancer and overexpressed hsa-miR-526b can downregulate the expression of Ku80, thus causing G0/G1 phase arrest and significantly suppressing the NSCLC growth in vitro and in vivo [68]. After the binding of the Ku70/Ku80 complex to the DSB, the catalytic subunit DNA-PKcs is recruited and activated. Active DNA-PK phosphorylates a variety of targets, including XRCC4, and together with XRCC4 forms the XRCC4-ligase IV complex to ligate DNA ends [69]. It has been reported that upregulation of miR-101 efficiently reduces the protein levels of DNA-PKcs and ATM via binding to the 3′-UTR of DNA-PKcs mRNA and inhibiting transcription. In this regard, miR-101 can sensitize the tumor cells to radiation in vitro and in vivo by hindering both NHEJ repair and ATM-mediated signal transduction [23].

Unlike the error-prone repair mechanism NHEJ, HR is an error-free pathway that functions only in the late S and G2 phases of the cell cycle as it requires a homologous sequence located on the sister chromatid. HR is initiated by CtIP and is promoted by various proteins, including the MRN complex. RAD51 mediates the invasion of the damaged DNA strand into the undamaged DNA duplex of the sister chromatid. A variety of miRNAs have been found to mediate chemosensitization by impacting RAD51 expression in HR repair mechanism. miR-107 was shown to directly target RAD51 and RAD51D and expression level correlated with PARP inhibitor sensitivity [35,70]. Overexpression of miR-103, miR-222, and miR-96, but not miR-107, decrease RAD51 expression and efficiency of HR repair [35,36,70]. BRCA1, a tumor suppressor gene, be recruited at DNA lesions and facilitates DNA repair and cell-cycle regulation as part of the large multisubunit BRCA1-associated genome surveillance complex (BASC). miR-182 expression can impact DNA repair and cellular sensitivity to PARP inhibitors by targeting BRCA1 mRNA and inhibiting its protein expression [30]. miR-99 can also mediate HR repair by regulating its target, SNF2H, which is required for recruitment of BRCA1 and RAD51 to sites of breaks. Therefore, upregulation of miR-99a reduced DSB repair following IR through repression of SNF2H [71].

### 2.3. Effectors of Apoptosis and Cell Cycle Checkpoint

Tumor cells often exhibit at least one cell cycle checkpoint defect. Therefore, inhibiting the transition of other remaining checkpoints should prevent cell cycle progression and reduce DNA damage repair time, resulting in more tumor cells killed by radiotherapy or chemotherapy [72]. DNA-damage-induced G2/M checkpoints are controlled by a p53-dependent or a p53-independent pathway. In the p53-independent pathway, CHK1/CHK2 blocks activation of cyclinB/CDK1 and causes G2 arrest by controlling the phosphorylation or activity of CDC25A, CDC25B, CDC25C, PLK1, and WEE1. Recently, it was reported that inhibition of the miR-15 family failed to increase radioresistance in breast cancer cells. The miR-15 family has been verified to negatively regulate CHK1 and WEE1 at both the mRNA and protein levels, and reduction of CHK1 and WEE1 subsequently prolonged γ-H2AX expression after irradiation, which increased radiosensitivity of cancer cells. In addition to the impact on γ-H2AX expression, inhibition of WEE1 and CHK1 effectively abrogates G2 arrest [62]. Overexpression of miR-497 and miR-890 can also affect the DDR and enhance cellular sensitivity to cisplatin and IR by promoting WEE1 expression [42,44].

The tumor suppressor p53 plays a central role in both DNA damage-induced G1/S and G2/M checkpoints. ATM/CHK2 or ATR/CHK1 (CHEK1) phosphorylate and stabilize p53, which in turn upregulates p21 to suppress the activity of the cyclin E(A)/CDK2 complex or the cyclinB/CDK1 complex, leading to G1 or G2 arrest. In addition to its extensive function in regulation of cell cycle checkpoints, p53 also plays a critical role in DSB-induced apoptosis. If DNA damage is not repaired in a timely manner, cells will initiate apoptosis before the DNA lesions enlarge and lead to more serious consequences, such as cancers. In response to DSBs, ATM/CHEK2 and ATR/CHK1 (CHEK1) phosphorylate and stabilize p53, resulting in transcriptional activation of pro-apoptotic factors, such as FAS, PUMA, and BAX [15]. Recent studies have determined that several miRNAs are involved in posttranscriptional regulation of p53 by directly binding to the p53 3′-UTR. miR-25 and miR-30d interact with the 3′-UTR of the human p53 gene and negatively regulate p53 expression. Downregulation of p53 leads to suppression of its target genes, such as p21, BAX and Puma, reducing G1 arrest and apoptosis [73]. Overexpression of miR-504 can mediate apoptosis and cell-cycle arrest in response to stress by downregulating p53 expression by binding to two sites in the p53 3′-UTR [46]. Moreover, miR-7 blocked SMARCD1 expression by binding to two seed regions in the 3′-UTR of SMARCD1 and downregulated SMARCD1 mRNA expression. With chemotherapy, miR-7 downregulated p53-dependent apoptosis-related genes BAX and p21 by interfering with the interaction of SMARCD1 and p53, thereby reducing caspase-3 cleavage and the downstream apoptosis cascades [74].

By disturbed miRNAs expression and further affect cell cycle checkpoint- or apoptosis-related targets expression, tumor cellular radio- or chemo-sensitivity could be changed, which is in favor of efficient and specific tumor therapy.

## 3. miRNA’s Therapeutic Function in Cancer Based on Its Regulation of the DDR

### 3.1. DDR-Related miRNAs as Biomarkers in Clinical Cancer Therapy

miRNAs have emerged as highly tissue-specific biomarkers, and much evidence shows that miRNA profiles are cell- and tumor-specific [75,76]. For example, expression of miR-155 is significantly higher in lung cancer tissues than in paracancerous and normal tissues [64]. In addition, the average expression level of miR-143 is downregulated in nasopharyngeal carcinoma cells and clinical specimens [77]. Some of these miRNAs that are differentially expressed in tumorous and healthy tissues are reported to target DDR-related genes and regulate cellular DDR processes. miR-338-5p is significantly downregulated in glioblastoma multiforme tumor samples compared with control tissue, and increased cell cycle arrest and apoptosis are observed in miR-338-5p-overexpressing cells [78]. A high level of miR-155 expression in lung cancer cells caused downregulation of expression levels of caspase-9 and Bax, which are the mitochondrial apoptotic pathway proteins [64]. In addition, as an important gene in the HR DNA repair pathway, RAD51 is also targeted by miR-155, and analysis of clinical data for triple negative breast cancer shows that low miR-155 expression level correlates with worse progression-free survival [37]. Moreover, DDR-related miRNA expression levels differ between drug-sensitive and -resistant cancer cells. miR-203 is upregulated in the oxaliplatin-resistant colorectal cancer cell (CRC) lines HT29, HCT116, and RKO. miR-203 regulates a kinase important in the DDR process and ATM expression by binding to its 3′-UTR [24]. miR-18a and miR-31, but not miR-203, are both expressed at a higher level in radiosensitive than in radioresistant cells [79,80]. Hence, the identification of altered DDR-related miRNAs affecting the therapeutic response may be helpful in determining the optimal and alternative treatments for cancer patients.

The miR-17-92 cluster has been shown to regulate various cellular processes, including apoptosis and DNA-damage signal transduction [81]. miR-92a is a member of the miR-17-92 cluster. Ohyashiki and colleagues evaluated the clinical relevance of miR-92a in plasma obtained from non-Hodgkin lymphoma patients, and observed that plasma miR-92a values in NHL patients were extremely low, compared with healthy subjects [82]. However, after complete remission in response to chemotherapy (rituximab plus cyclophosphamide, doxorubicin, vincristine and prednisone (CHOP) or CHOP-like regimens), the very low plasma level of miR-92a in NHL patients was increased slightly. Interestingly, the levels of miR-92a decreased again in patients with relapsed disease, suggesting that plasma miR-92a levels correlate with disease conditions in lymphoma patients, and plasma levels of miR-92a might be a useful marker to evaluate therapeutic efficiency or predict tumor recurrence [82].

Overexpression of another DDR-related miRNA, miR-155, increased with progression from normal B cells to monoclonal B-cell lymphocytosis (MBL) to overt chronic lymphocytic leukemia (CLL), and miR-155 expression levels were significantly higher in patients who failed to achieve a complete response than in those who experienced a complete response. Those findings support the use of cellular and plasma levels of miR-155 as biomarkers of the risk of progression in individuals with MBL, as well as to identify patients with CLL who may not respond well to therapy [83].

### 3.2. DDR-Related miRNAs as Sensitizers to Radiotherapy or Chemotherapy

The most common way of treating cancer is to expose the body to agents (including radiotherapy and chemotherapy) that kill cancer cells more efficiently than normal cells. Many cancer drugs employed in the clinic have been used for several decades, and the efficacy of anticancer drugs is highly influenced by the cellular DDR process, whereas recurrent resistance to drug treatments in cancer cells is a key barrier to therapeutic efficiency. Drug-resistant cancer cells can proliferate exponentially, become more aggressive, and have a higher incidence of aggressive metastasis to other organs. Drug resistance is classified in two ways. The first of these is intrinsic resistance, whereby tumors are resistant prior to treatment and, therefore, the drugs do not effectively treat the tumor even with initial early diagnosis and treatment. Another form of resistance is acquired resistance, which occurs despite an initial positive response to therapy [84]. Because of the key role of miRNAs in targeting and regulating multiple genes involved in the DDR, modulation of endogenous miRNA expression may be a very attractive strategy to overcome radio-/chemoresistance. The most important miRNAs and their target DDR genes involved in anticancer drug response are listed in Table 1.

In the previous section, we pointed out that miR-203, miR-18a, and miR-31 are all differentially expressed in sensitive and resistant cells. Further, several DDR-related miRNAs were found to be downregulated and to modulate sensitivity to chemotherapy. miR-152 expression was dramatically downregulated in the cisplatin-resistant cell lines A2780/CP70 and SKOV3/DDP compared with their respective parental cells. Overexpression of miR-152 sensitized cisplatin-resistant ovarian cancer cells by reducing cisplatin-induced autophagy and enhancing cisplatin-induced apoptosis and inhibition of cell proliferation [85]. Another study also found reduced expression of miR-15 family members (miR-15a, miR-15b, miR-16, miR-195, miR-424, and miR-497) and miR-155 in cisplatin-resistant cells. Ectopic expression of the two miRNAs sensitizes the cells to cisplatin-induced apoptosis by targeting WEE1 and CHK1 (CHEK1) kinases [43]. In addition to sensitivity to chemotherapy, specific miRNAs that sensitize or protect cells from radiation have also been reported recently. For instance, miR-205 is downregulated in radioresistant subpopulations of breast cancer cells. Because of a positive correlation between miR-205 expression and radiosensitivity, therapeutic delivery of miR-205 mimics via nanoliposomes in a xenograft modelsensitized the tumor to radiation [40]. These results highlight a potential strategy whereby miRNA levels may be altered by administration of exogenous agents, such as mimics or anti-miRs, to sensitize cancer cells to radio-/chemotherapy and overcome resistance.

Although miRNA expression profiles differ between resistant and sensitive cancers, environmental stressors can influence miRNA expression in any cancer type. Many studies have revealed that expression levels of several miRNAs change significantly upon radiotherapy or chemotherapy, and do so reproducibly across various cell types. For example, almost all of the miR-15 family miRNAs (miR-15a/b, miR-195, miR-424, and miR-497) were downregulated across a number of different cell lines, including endothelial cells, non-small cell lung cancer (NSCLC) cells, and lymphoblasts [97], whereas miR-148b was repressed by IR in endothelial cells, but induced by IR in non-Hodgkin lymphoma [98,99]. Moreover, upregulation of miR-21 is observed in both IR- and doxycycline-stimulated cells [65,100]. The variability in drug-induced changes in expression suggests that miRNA expression is modified with exposure to anticancer drugs, and expression levels affect the response to anticancer drugs based on their involvement in DDR-regulatory mechanisms at different levels and through many pathways.

Acting as tumor suppressors, let-7 family miRNAs strongly contribute to the regulation of DDR and cell proliferation and are expressed in many tumors [101]. Weidhaas’ and other groups have revealed that the expression of let-7 miRNAs is modified upon irradiation in various cancer cells, such as glioblastoma and NSCLC cells [102,103,104]. The modified expression of let-7 miRNAs following IR is dependent on p53, which is a key protein in the IR-activated ATM signaling pathway [105]. Further, miR-34a, which is upregulated after exposure to IR or etoposide, is another p53 target. miR-34a functions downstream of the p53 pathway as a tumor suppressor and is involved in regulating cell cycle arrest and apoptosis [106,107]. In addition to miR-34, it is well established that miR-21 targets many genes (Cdc25A, pAKt, PTEN) in the cell cycle arrest, DDR, and apoptotic pathways, and is consistently upregulated upon IR in a variety of normal and cancer cell lines [108,109].

Consistent with the intimate connection between DDR-related miRNAs and drug resistance, the combined use of DDR-related miRNAs and radio- or chemotherapy has been demonstrated to increase therapeutic efficiency. It has been reported that, by encapsulating miR-34a mimics with liposomes and delivering it into mice, the levels of miR-34a in tumors were increased and the mRNA levels of several miR-34a targets were decreased [110]. In addition, Conde and coworkers have successfully targeted and silenced miR-21 expression by Gold-nanobeacon in pancreatic cancer cell [111]. Moreover, intravenously delivered miR-16 mimics are also currently under Phase I clinical trials for patients with MPM (Malignant Pleural Mesothelioma) and NSCLC (Non-Small Cell Lung Cancer), and preliminary data show manageable safety profile in five patients [112]. Thus, extraneously changing the level of miRNAs can be realized, and miRNA-based therapy might bring new options to cancer treatment.

## 4. Conclusions

In summary, miRNAs are involved in regulating almost every aspect of the DDR; even only one miRNA can mediate multiple targets in the DDR signaling pathway, DNA repair, cell cycle arrest, and apoptosis. This means that miRNA-mediated DDR is a significant and complicated regulatory process. Finding the significant role of miRNAs in DDR and radio- or chemotherapy may provide a new way for us to use miRNAs as potential tools, biomarkers or sensitizers in cancer treatment. Nevertheless, because of the complicated network regulating the interactions of miRNAs and their target genes, much research is needed in the future to clarify the correlation between miRNAs and the DDR.

## Figures and Tables

**Table 1:** Summary of miRNAs and their target DDR genes involved in response to anticancer treatment.

Cancer	miRNA	Target(s)	Therapy	Effects	References
Breast	miR-18a	ATM	IR	Radiosensitivity	[21]
	miR-155	RAD51	IR	Radiosensitivity	[37]
	miR-107	RAD51	PARP inhibitor	Chemosensitivity	[35]
	miR-182	BRCA1	IR	Radiosensitivity	[30]
	miR-34a	Bcl-2	Docetaxel	Chemoresistance	[86]
	miR-21	PTEN and PDCD4	Chemotherapy	Chemoresistance	[87]
	miR-125b	BAK1	Paclitaxel	Chemoresistance	[47]
Lung	miR-138	H2AX	IR	Radiosenstivity	[19]
	miR-101	ATM	IR	Radiosensitivity	[88]
	miR-497	Bcl-2	Chemotherapy	Chemosensitivity	[89]
	miR-34a	RAD51	IR	Radiosensivity	[33]
	miR-155	Apaf-1	Chemotherapy	Chemorisistance	[64]
	miR-141	PDCD4	Cisplatin	Chemoresistance	[90]
Ovarian	miR-506	RAD51	Chemotherapy	Chemosensitivity	[34]
	miR-152	ATG14	Cisplatin	Chemosensitivity	[85]
	miR-31	KCNMA1	Cisplatin	Chemoresistance	[91]
Hepatoma	miR-16	Bcl-2	Epigallocatechin gallate	Chemosensitivity	[61]
	miR-210	AIFM3	IR	Radioresistance	[92]
Colorectal	miR-145	RAD18	5-FU	Chemosensitivity	[32]
	miR-203	ATM	Oxaliplatin	Chemoresistance	[24]
Glioblastoma	miR-100	ATM	IR	Radioresistance	[93]
	miR-338-5P	RHEB	IR	Radiosensitivity	[78]
	miR-181b	SENP2	IR	Radioresistance	[87]
Renal carcinoma	miR-185	ATR	IR	Radiosensitivity	[27]
Prostate	miR-744-3P	RAD23B	IR	Radiosensitivity	[42]
	miR-890	MAD2L2, WEE1, XPC	IR	Radiosensitivity	[42]
Cervical	miR-145	HLTF	IR	Radiosensitivity	[94]
Bladder	miR-193a-3P	HOXC9	Chemotherapy	Chemoresistance	[95]
Hematopoietic	miR-24	H2AX	Cisplatin	Chemosensitivity	[96]
